# Physical activity, aerobic fitness, and AD blood biomarkers: The IGNITE study

**DOI:** 10.1002/alz.71484

**Published:** 2026-05-18

**Authors:** Lauren E. Oberlin, Patricio Solis‐Urra, Kelsey R. Sewell, Audrey M. Collins, Chaeryon Kang, Haiqing Huang, George Grove, Lu Wan, Arthur F. Kramer, Edward McAuley, Jeffrey M. Burns, Charles H. Hillman, Eric D. Vidoni, Anna L. Marsland, M. Ilyas Kamboh, Amanda Szabo‐Reed, Renee J. Rogers, Jill Morris, Xuemei Zeng, Thomas K. Karikari, John M. Jakicic, Kirk I. Erickson

**Affiliations:** ^1^ Neuroscience Institute AdventHealth Research Institute Orlando Florida USA; ^2^ Department of Psychiatry Weill Cornell Medicine New York New York USA; ^3^ Faculty of Education and Social Sciences Universidad Andres Bello Viña del Mar Chile; ^4^ Centre for Healthy Ageing, Health Futures Institute Murdoch University Murdoch Western Australia Australia; ^5^ Department of Psychiatry University of Pittsburgh Pittsburgh Pennsylvania USA; ^6^ Department of Biostatistics and Health Data Science University of Pittsburgh Pittsburgh Pennsylvania USA; ^7^ Department of Psychology University of Pittsburgh Pittsburgh Pennsylvania USA; ^8^ Beckman Institute for Advanced Science and Technology University of Illinois at Urbana Champaign Urbana Illinois USA; ^9^ Department of Health and Kinesiology University of Illinois at Urbana‐Champaign Urbana Illinois USA; ^10^ Alzheimer's Disease Research Center University of Kansas Medical Center Kansas City Kansas USA; ^11^ Institute for Cognitive and Brain Health Northeastern University Boston Massachusetts USA; ^12^ Department of Psychology Northeastern University Boston Massachusetts USA; ^13^ Department of Physical Therapy, Movement, and Rehabilitation Sciences Northeastern University Boston Massachusetts USA; ^14^ Department of Human Genetics University of Pittsburgh Pittsburgh Pennsylvania USA; ^15^ Department of Internal Medicine, Division of Physical Activity and Weight Management University of Kansas Medical Center Kansas City Kansas USA; ^16^ Biofluid Biomarker Laboratory, Western Psychiatric Hospital University of Pittsburgh Medical Center Pittsburgh Pennsylvania USA; ^17^ Alzheimer's Disease Research Center University of Pittsburgh Pittsburgh Pennsylvania USA

**Keywords:** aerobic fitness, amyloid positron emission tomography, cognition, episodic memory, executive function, modifiable risk factor, neuropathology, physical activity, plasma biomarkers, preclinical Alzheimer's disease

## Abstract

**INTRODUCTION:**

Physical activity (PA) and cardiorespiratory fitness (CRF) are associated with reduced risk of cognitive decline and dementia, yet their relationships with dementia‐related pathophysiology remain unclear. In a community‐dwelling older adult cohort, we examined associations between objectively measured PA, CRF, biomarkers of Alzheimer's disease (AD)‐related pathology, and cognition.

**METHODS:**

Participants (*n* = 648, 71% female, age 69.88 ± 3.75) completed a comprehensive cognitive evaluation, objective assessments of moderate‐to‐vigorous PA (MVPA) and CRF (VO_2peak_), and AD‐related brain (positron emission tomography [PET] amyloid beta [Aβ]) and blood biomarkers (Aβ1–42/1–40, phosphorylated tau [p‐tau]217, p‐tau181, glial fibrillary acidic protein [GFAP], neurofilament light chain [NfL]).

**RESULTS:**

Greater MVPA (β = −0.107; *p* = 0.013) and CRF (β = −0.114; *p* = 0.027) were associated with lower NfL, but not Aβ PET, p‐tau217, Aβ1–42/1–40, or GFAP. Aβ positivity moderated the CRF–NfL relationship, with higher CRF linked to lower NfL specifically among Aβ‐positive individuals. NfL mediated relationships between MVPA, CRF, and cognitive performance in select domains.

**DISCUSSION:**

Neuroprotective benefits of PA may be conferred through mechanisms influencing neurodegeneration, particularly among those with emerging AD pathology.

## Highlights

- Greater objectively measured physical activity was associated with lower neurofilament light chain (NfL) levels.
- Physical activity was unrelated to biomarkers of core Alzheimer's disease pathology (amyloid beta [Aβ] positron emission tomography, phosphorylated tau 217).
- NfL mediated associations between physical activity and cognition in select domains.
- Similar patterns were found for cardiorespiratory fitness (CRF), a physiological measure of aerobic fitness.
- Higher CRF was related to lower NfL specifically among Aβ–positive individuals.

## 1 BACKGROUND

The neuropathologic changes in Alzheimer's disease (AD) may begin decades prior to clinical onset, suggesting opportunity for early intervention and prevention.^1^ Lifestyle factors such as physical activity (PA) may attenuate cognitive decline, lower dementia risk, and delay clinical progression.^2^, ^3^ However, the disease‐modifying effects of PA remain unclear. A key unresolved question is whether and how PA relates to core pathogenic markers of AD, particularly prior to clinical onset. Characterizing the molecular mechanisms of PA may help direct new preventive approaches and facilitate more precise implementation of behavioral interventions.

Animal models suggest that PA may reduce AD risk by directly modulating core disease pathology.^4^, ^5^ However, translational evidence in humans is mixed and inconsistent.^6^ While some studies show inverse relationships between PA and amyloid beta (Aβ) or tau measured via cerebrospinal fluid or positron emission tomography (PET) imaging,^7^, ^8^ others fail to find these associations.^6^, ^9^, ^10^ Recent advances in blood biomarkers of AD‐related pathology offer an opportunity to enhance understanding of the neuropathologic processes potentially modified by PA. Specifically, plasma Aβ1–42/1–40, phosphorylated tau (p‐tau) 181 and p‐tau217 capture core AD pathology,^11^, ^12^ with p‐tau217 demonstrating the highest correspondence with brain Aβ.^11^ AD pathogenesis also involves neurodegeneration and astrocytic activation, measured via neurofilament light chain (NfL) and glial fibrillary acidic protein (GFAP)^13^, ^14^—non‐specific markers shown to improve detection and prognostic assessment of AD.

Despite recent progress in plasma biomarkers, research leveraging these markers to evaluate links between PA and AD‐related pathology is limited. Existing studies are few in number, often investigate a narrow subset of biomarkers, and demonstrate mixed results.^15^, ^16^, ^17^, ^18^, ^19^ For example, several studies have found that greater self‐reported PA relates to lower NfL concentrations,^20^, ^21^ but not Aβ1–42/1–40 or p‐tau181.^21^, ^22^, ^23^ In contrast, other studies report that higher self‐reported PA relates to lower total tau levels^16^ and slower longitudinal increases in p‐tau181.^17^ Additionally, research examining PA associations with newer biomarkers that show improved accuracy for reflecting neuropathology (e.g., p‐tau217) is limited, and few studies have assessed relationships to cognition, particularly across multiple cognitive domains. Finally, while PA is considered a promising modifiable target for dementia prevention, limited research in those without clinical impairment restricts understanding of how PA may influence early disease processes and mitigate future risk.

**RESEARCH IN CONTEXT**

1. **Systematic review**: Studies identified in a Google Scholar search to October 2025 report mixed findings regarding associations between physical activity, aerobic fitness, and blood biomarkers of Alzheimer's disease (AD) pathology. Prior studies have been largely limited to self‐reported activity measures, samples with clinical impairment, and limited scope of blood biomarkers, with few examining potential clinical moderators.
2. **Interpretation**: Both physical activity and fitness were selectively associated with a general marker of neurodegeneration, while no relationships were observed with brain or blood biomarkers of core AD pathology. A novel moderation by amyloid beta positivity was observed, indicating that maintaining higher fitness may be a promising approach to mitigate neurodegenerative changes in those with preclinical AD pathology.
3. **Future directions**: Prospective studies are needed to clarify how changes in physical activity and fitness may impact disease pathology, and determine the ideal timing, dose, and intensity of physical activity and personalized fitness recommendations to advance strategic prevention efforts.

Another key factor limiting understanding of the relationship between PA and AD‐related pathology is the broad use of self‐report measures of PA,^15^, ^16^, ^17^, ^19^, ^21^, ^22^, ^23^ which lack precision and are often subject to bias and overreporting. Objective measures like accelerometry provide more precise and reliable estimates of PA and may better capture associations with disease pathology, yet just one prior study (*N* = 242) has leveraged objective PA data to evaluate relationships with blood biomarkers.^24^ Reliance on self‐report measures, frequent stratification of self‐report measures into arbitrary groups (e.g., high vs. low activity), small sample sizes, and assessment of biomarkers with limited sensitivity to AD pathology (e.g., Aβ1–42/1–40, total tau) may be contributing to the inconsistent findings in prior work. In addition to PA behavior, cardiorespiratory fitness (CRF) can be estimated to provide an objective physiological index of aerobic capacity. CRF, which can be improved by habitual PA, is a robust predictor of overall longevity^25^ and has been linked to reduced risk of cognitive decline and dementia,^26^, ^27^, ^28^, ^29^, ^30^ though only one prior study has evaluated CRF associations with blood biomarkers.^9^ To address these gaps, we leveraged data from a large community‐dwelling cohort with objective measures of PA, CRF, and multiple validated AD biomarkers to further characterize associations with PA and identify potential pathways linking PA to cognitive health in cognitively unimpaired older adults.

This study examined cross‐sectional relationships between objectively measured PA, CRF, and brain and fluid measures of AD‐related pathology, including those that are disease specific (brain Aβ PET, Aβ1–42/1–40, p‐tau181, p‐tau217) and those representative of multiple pathways (GFAP, NfL). Using a large multicenter sample of cognitively unimpaired older adults, we also evaluated whether demographic (sex) and clinical factors (apolipoprotein E [*APOE*] ε4 carriage, Aβ positivity) moderated associations of PA and fitness with blood biomarkers. Given prior findings demonstrating positive associations among PA, CRF, and cognition in this sample,^26^, ^31^ we further tested whether blood biomarkers would statistically mediate associations with cognition across a range of domains. We hypothesized that higher PA and CRF would associate with general rather than disease‐specific markers of neuropathology, and that PA‐related variability in select biomarkers would further relate to better performance across multiple cognitive domains.

## 2 METHODS

### 2.1 Participants

Adults aged 65 to 80 years were recruited for participation in a 12‐month multi‐site randomized clinical trial assessing the impact of exercise on cognition and brain health (Investigating Gains in Neurocognition in an Intervention Trial of Exercise [IGNITE]; ClinicalTrials.gov: NCT02875301). Baseline data were used for the current analyses. Participants were excluded if they had history or presence of neurological conditions (e.g., Parkinson's disease, stroke, dementia), current major depression, substance use disorder in the past 5 years, history of severe mental illness (e.g., schizophrenia), self‐reported engagement in > 20 minutes per day of structured moderate‐to‐vigorous intensity PA ≥ 3 days per week over the past 6 months, recent history of severe cardiovascular events (e.g., congestive heart failure, angioplasty), Type I diabetes, uncontrolled or insulin‐dependent Type II diabetes, or magnetic resonance imaging contraindications. The protocol and full eligibility criteria are detailed in Erickson et al.^32^

A total of 648 participants were enrolled in IGNITE, including 357 participants with PET Aβ data (obtained between September 2017 and December 2020) collected across three sites: the University of Pittsburgh, Northeastern University, and University of Kansas Medical Center. The demographic composition of the study sample reflected the regional demographic representation of racial and ethnic minorities at each of the three study sites, according to US Census data. Participants self‐identified their race and ethnicity along the National Institutes of Health guidelines. The study was approved by the institutional review board at each site with the following protocol numbers: Pittsburgh (STUDY19110244), Kansas (STUDY00140896), and Northeastern (17‐05‐02). All participants provided written informed consent before data collection.

### 2.2 Cognitive assessment

Baseline cognitive function was assessed through a comprehensive neuropsychological battery, consisting of paper‐and‐pencil and computerized assessments delivered across 2 days by annually certified psychometricians. The assessment battery included measures of processing speed (Letter Comparison Test, Digit Symbol Substitution Test, Trail Making Test, Part A), episodic memory (Brief Visuospatial Memory Test, Picture Sequencing Test, Hopkins Verbal Learning Test, Logical Memory Task, Montreal Cognitive Assessment [MoCA] free recall, Verbal Paired Associates), working memory (N‐Back Working Memory Task, Spatial Working Memory Task, List Sorting Working Memory Task), visuospatial abilities (Matrix Reasoning, Spatial Relations, MoCA Clock Draw), and executive function (EF)/attentional control (Flanker Task, Stroop Task, Dimensional Change Card Sort task, Trail Making Test, Part B). Performance in each domain was measured using previously established latent factors, ^26^ with higher values reflecting better performance. Additional details of the cognitive assessment are reported elsewhere.^26^, ^32^

### 2.3 Physical activity assessment

Accelerometry was used to objectively measure PA. Triaxial accelerometers (ActiGraph GT9X Link; Ametris [formerly ActiGraph]) were initialized at 70 Hertz and worn on the non‐dominant wrist for seven continuous days. Raw data were processed using GGIR (version 2.10‐1).^33^ A Euclidean Norm Minus One with negative values rounded to zero (ENMO) acceleration metric was used.^34^ Epochs were aggregated to 60 seconds in part 5, using acceleration‐based cut points to classify behaviors as follows: < 35 milligravity (m*g*) as sedentary behavior; 35 to 99 m*g* as light PA; ≥ 100 m*g* as moderate‐to‐vigorous PA (MVPA) during the waking period. The sensor needed to be worn for at least 16 hours (and two thirds of waking hours) to be considered a valid wear day. Midnight‐to‐midnight was used to distinguish each 24‐hour period. MVPA served as the primary predictor in statistical models, and was calculated as the plain average of minutes per day spent in MVPA, averaged across valid wear days. Sensitivity analyses were performed excluding participants with minimal or excessive accelerometer wear time (*n* = 30), defined as < 4 valid wear days (including < 3 weekdays and < 1 weekend day) or > 10 total days, respectively. Extensive quality control procedures were performed to assess artifacts, calibration errors, and compliance with the protocol, as previously described.^35^ Of the 648 participants enrolled in the IGNITE study, 26 subjects were missing actigraphy data at baseline, and 33 subjects were removed through quality control procedures. Please see Collins et al.^31^ and Sewell et al.^35^ for additional details.

### 2.4 CRF assessment

CRF was assessed by trained exercise physiology staff using a graded exercise test performed on a motorized treadmill. To determine treadmill speed, participants walked at an agreed‐upon pace between 1.5 to 3.5 mph that resulted in a heart rate of 70% of age‐predicted maximal heart rate (APMHR) ± 5 beats. For anyone reporting use of beta blocker medications, a rating of perceived exertion (RPE) of 11 on the Borg rating scale^36^ was used. Participants demonstrating American College of Sports Medicine (ACSM)‐defined contraindications for completing a graded exercise test were not permitted to continue the test.^37^

The graded exercise test started with a 1‐minute standing rest phase followed by a 2‐minute warm‐up phase walking at 0.50 mph less than the agreed‐upon test speed with zero incline. Treadmill speed was maintained throughout the test but a 2% increase in grade was executed every 2 minutes, using a modified Balke protocol.^38^ During each stage, electrocardiography was monitored and blood pressure and RPE were obtained. Prior to each VO_2_ test, the metabolic cart was calibrated with a three liter syringe at room temperature along with gas calibration with known levels of oxygen, carbon dioxide, and nitrogen. Manufacturer‐recommended quality control was also performed every 6 months. Throughout the test, oxygen consumption was measured continuously using indirect calorimetry with metabolic carts (Parvo Medics TrueOne 2400; COSMED Quark CPET). The test was completed to volitional exhaustion or with symptom limitation, and was followed by a 4‐minute active cooldown and a 4‐minute resting cooldown. The highest VO_2_ value obtained during the test was used as the indicator of VO_2peak_ and represents the measure of CRF used here. We also recorded whether the test met standard ACSM criteria^37^ for defining maximal effort on a graded exercise test including: (1) plateau in VO_2_ between two or more workloads (increase < 0.15L/minute or 2.0 mL/kg/minute during the last minute of corresponding workloads), (2) respiratory exchange ratio (RER) ≥ 1.10, (3) heart rate within 10 beats of the APMHR (220‐age), and (4) an RPE ≥ 17. Maximal effort is typically defined as achieving at least three out of these four criteria.^37^

### 2.5 Blood biomarkers

Approximately 47 cc of fasted blood was collected between 8:00 am and 10:00 am. Aβ1–42 and Aβ1–40 levels were measured using immunoprecipitation‐mass spectrometry (IP‐MS)^39^ and used to calculate the Aβ1–42/1–40 ratio. The Simoa platform (Quanterix) was used to measure p‐tau181, p‐tau217, NfL, and GFAP, as previously described.^40^, ^41^ P‐tau217 was measured using the ALZpath assay kit (#104371), and p‐tau181 using the V2 Advantage kit (#103714) at the University of Pittsburgh. NfL and GFAP were analyzed using the N2PB assay (#103520) on a Quanterix‐HD‐X at the University of Kansas Medical Center.^40^, ^41^ To evaluate reproducibility, quality control samples were assessed at the beginning and the end of each run. The average within‐run coefficients of variation (CVs) were: p‐tau181 = 6.3%, p‐tau217 = 11.0%, NfL = 6.3%, and GFAP = 9.7%. The mean between‐run CVs were p‐tau181 = 10.3%, p‐tau217 = 11.4%, NfL = 8%, and GFAP = 14.8%. Further details about assay performance and validation can be found in Sewell et al.^41^ and Olvera‐Rojas et al.^40^

### 2.6 Amyloid PET

Consistent with Alzheimer's Disease Neuroimaging Initiative (ADNI) protocols,^42^ each participant in the PET subsample (*N* = 357) completed a 20 minute PET scan (4*5 minutes) beginning at 90 (± 5) minutes after intravenous injection of 8.0 mCi ± 20% of florbetaben (Neuraceq) followed by 10 mL normal saline flush. PET images were processed using SPM12 in MATLAB. PET images were motion corrected and averaged across four volumes, then registered to the participant's T1‐weighted magnetization‐prepared rapid gradient echo image. Voxel‐wise standard uptake value images were calculated for each motion‐corrected PET image. Whole‐brain standardized uptake value ratios were generated using the whole cerebellum as the reference region and standard volumes of interest, and subsequently converted to a Centiloid scale using established methods.^42^

### 2.7 Covariates and moderators

Health and demographic factors that have been associated with variables of interest were included as covariates in statistical models, including chronological age (in years), sex (male/female), waist circumference, and *APOE* ε4 carriage. In addition, study site (Pittsburgh, Kansas City, Boston) was adjusted for in all statistical models, and formal years of education was included as a covariate in models containing cognitive outcome variables. Participants self‐reported date of birth, sex, and formal years of education. Anthropometric measures of body size have been shown to relate to blood biomarkers. ^43^, ^44^, ^45^ Waist circumference (cm), an anthropometric measure of body size and specifically a proxy of central adiposity, was measured horizontally at the midpoint between the lowest palpable rib and top of the iliac crest at the mid‐axillary line. Measurements were performed in duplicate to the nearest 0.1 cm using a Gulick spring‐loaded tape measure. Genotypes for the two *APOE* single nucleotide polymorphisms resulting in six genotypes was performed on blood samples using TaqMan assays.^46^ Participants with at least one *APOE* ε4 allele (ε2/ε4, ε3/ε4, and ε4/ε4 genotypes) were classified as *APOE* ε4 carriers.

### 2.8 Statistical analysis

Analyses were conducted using R version 4.5.0 (R Foundation for Statistical Computing). We assessed normality through visual inspection and skewness and kurtosis analysis. As previously described,^40^, ^41^ skewed variables (MVPA, p‐tau217, p‐tau181, NfL, GFAP) were log transformed prior to analyses. Outliers for fluid biomarkers were identified using a previously established approach,^40^, ^47^ which resulted in the following thresholds and participants removed: Aβ1–42/1–40 > 0.24 (*n *= 2); GFAP > 1010 pg/mL (*n *= 2); NFL > 76 pg/mL (*n *= 2); p‐tau217 > 2.16 pg/mL (*n *= 2); and p‐tau181 > 15 pg/mL (*n *= 1).

Primary linear regression models were used to assess associations between objectively measured MVPA and each AD‐related biomarker: Aβ PET Centiloid, Aβ1–42/1–40, p‐tau217, p‐tau181, NfL, and GFAP, adjusting for age, sex, *APOE* ε4 carriage, waist circumference, and study site. The significance threshold was set at *p* < 0.05. Data are presented as standardized betas and 95% confidence intervals, where both predictor and outcome variables were *z* scored. Secondary logistic regression models tested the association of MVPA with binarized variables of Aβ positivity, determined in the full cohort using a clinical cutoff of p‐tau217 ≥ 0.46 pg/ml, as previously defined in this sample,^40^ and a Centiloid threshold of ≥ 24 in the subsample with Aβ PET (*N* = 357).^11^ Only one prior study has examined associations of CRF with AD blood biomarkers, and thus we performed secondary linear regression analyses testing CRF (VO_2peak_) associations with AD‐related biomarkers. For biomarkers showing significant associations, we evaluated moderation by sex, *APOE* ε4 carriage, and Aβ positivity (per p‐tau217 cut‐off), and performed post hoc analysis of covariance models stratifying MVPA and CRF into quartiles to interrogate group differences.

Finally, for biomarkers that demonstrated significant associations with MVPA or CRF, exploratory analyses were performed using the R mediation package to examine whether AD‐related blood biomarkers statistically mediated associations between MVPA or CRF (predictors) and cognitive function (outcome). MVPA and CRF were included as independent variables in separate mediation models, with cognitive performance in each domain (processing speed, working memory, EF/attentional control, episodic memory, visuospatial processing) as outcome variables, adjusting for age, sex, waist circumference, *APOE* ε4 carriage, study site, and years of education. The presence of statistical mediation was assessed using the bias corrected and accelerated (BCa) bootstrap confidence intervals (CIs) for the indirect effect, based on 5000 bootstrapped resamples. If the 95% CIs do not contain zero, it indicates a statistically significant mediation effect at a significance level of 0.05. Mediation was considered present when the CI did not include zero. The proportion mediated was calculated as the ratio of the indirect and total effect.

## 3 RESULTS

### 3.1 Participant characteristics

The full sample included 648 older adults (mean [standard deviation (SD)] age = 69.88 [3.75]) with 461 women (71.1%) and an average of 16.32 (SD = 2.21) years of education. As previously reported,^40^, ^41^ the biomarker sample sizes varied slightly due to different amounts of missing data and outliers for each biomarker. The sample size for each biomarker is provided in Table 1. A total of 589 participants had complete accelerometer data, with a mean of 31.25 (SD = 26.46) minutes of MVPA per day. The subsample with accelerometer data did not differ in demographic, clinical, cognitive, or biomarker characteristics from the full IGNITE sample (Table 1; see Collins et al.^31^ and Sewell et al.^35^ for further details). Summary statistics for the sample characteristics stratified by amyloid positivity and negativity can be found in Table S1 in supporting information.

**TABLE 1:** Descriptive characteristics.

Variable	*N*	Full sample *N* = 648^a^	Accelerometry subsample *N* = 589	*p* value^b^
Age, year	648	69.88 ± 3.75	69.79 ± 3.71	0.68
Education, year	648	16.32 ± 2.21	16.33 ± 2.21	0.92
Waist circumference, cm	648	98.20 ± 14.41	98.15 ± 14.46	0.95
VO_2_ peak (mL/kg/min)	648	21.68 ± 5.06	21.79 ± 5.12	0.71
MVPA (minutes/day)	589		31.25 ± 26.46	
Race, White *N* (%)	648	491 (76%)	455 (77%)	0.59
*APOE* ε4 carriage	640			0.89
*APOE* ε4 non‐carrier, *N* (%)		466 (73%)	420 (72%)	
*APOE* ε4 carrier, *N* (%)		174 (27%)	161 (28%)	
Sex, *N* %	648			0.84
Female		461 (71%)	415 (70%)	
Male		187 (29%)	174 (30%)	
Site, *N* %	648			0.65
Kansas		214 (33%)	203 (34%)	
Northeastern		215 (33%)	181 (31%)	
Pitt		219 (34%)	205 (35%)	
Episodic memory	648	0.00 ± 0.61	0.01 ± 0.61	0.75
Processing speed	648	0.00 ± 0.62	0.02 ± 0.62	0.58
Working memory	648	0.00 ± 0.54	0.02 ± 0.54	0.54
EF/attentional control	648	0.00 ± 0.59	0.02 ± 0.59	0.56
Visuospatial processing	648	0.00 ± 0.56	0.02 ± 0.56	0.61
PET Centiloid	357	8.11 ± 29.69	8.02 ± 29.19	0.97
NfL, pg/mL	605	16.81 ± 6.82	16.67 ± 6.68	0.73
GFAP, pg/mL	606	178.75 ± 96.77	177.71 ± 93.50	0.85
Aβ1‐42/1‐40 ratio	627	0.10 ± 0.01	0.10 ± 0.01	0.88
p‐tau181, pg/mL	627	2.83 ± 1.55	2.85 ± 1.59	0.83
p‐tau217, pg/mL	625	0.43 ± 0.28	0.43 ± 0.28	0.95
Aβ positive, *N* (%)^c^		181 (29%)	165 (29%)	

Notes: Unless otherwise specified, data are presented as mean ± standard deviation.

^a^ As previously reported, the biomarker sample sizes varied slightly due to different amounts of missing data and outliers for each biomarker. The sample size for each biomarker in the full cohort is provided in the left column. Eight subjects did not have *APOE* genotype data.

^b^ Results from Welch two‐sample *t* test and Pearson chi‐squared tests assessing for differences across variables between the full cohort and the subsample with accelerometry data.

^c^ A p‐tau217 threshold of 0.46 pg/mL was used to classify Aβ positivity. This cut‐point was established in this sample using area under the curve analysis and the Youden index, as previously described.

Abbreviations: Aβ, amyloid beta; *APOE*, apolipoprotein E; EF, executive function; GFAP, glial fibrillary acidic protein; MVPA, moderate‐to‐vigorous physical activity; NfL, neurofilament light chain; PET, positron emission tomography; p‐tau, phosphorylated tau.

### 3.2 PA, CRF, and AD‐related biomarkers

**FIGURE 1:**
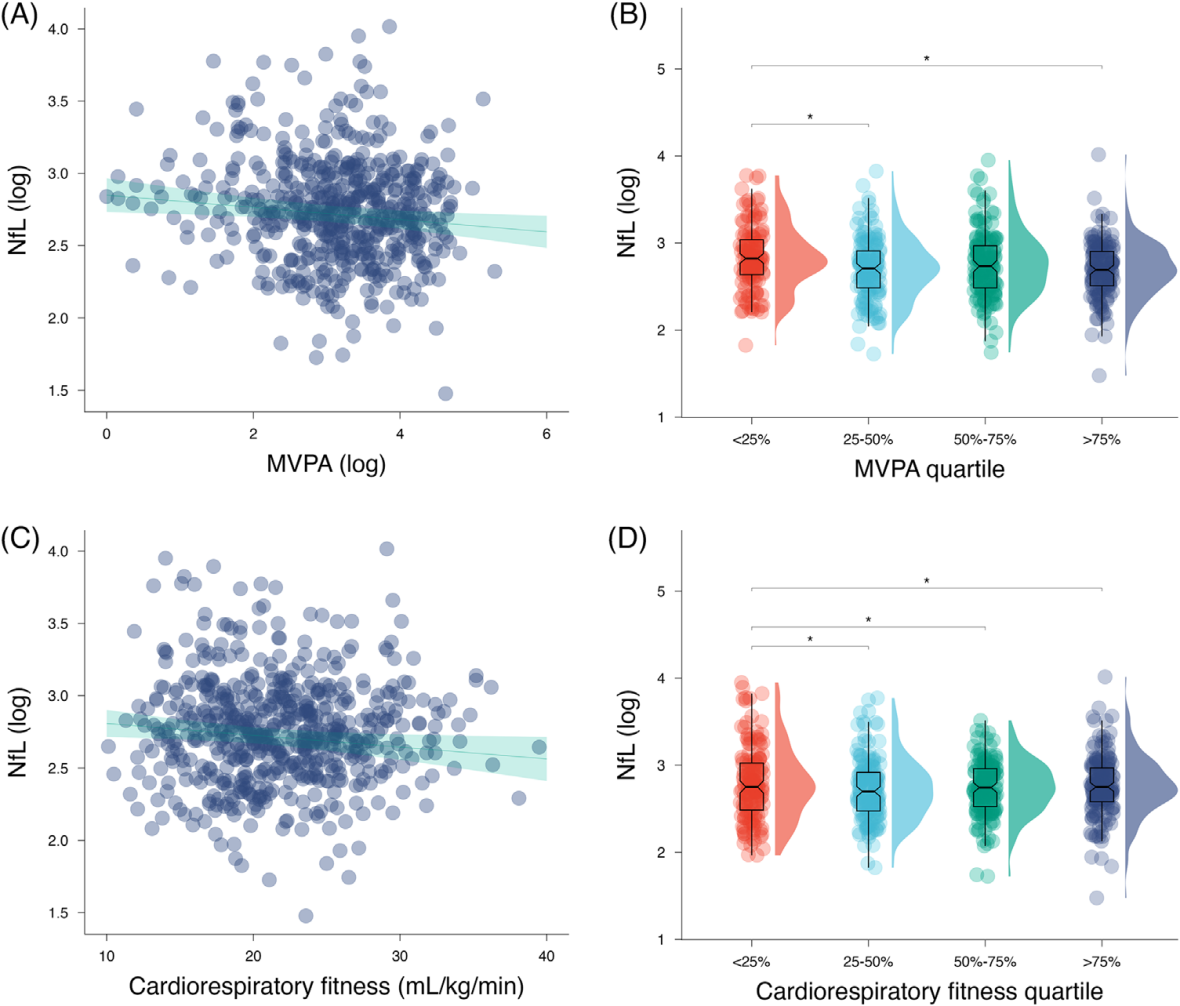
Associations among MVPA, CRF, and NfL concentrations. Scatterplot depicting associations between objectively measured MVPA (A) and CRF (C) with NfL. Shaded areas reflect 95% confidence intervals. (B) and (D) depict concentrations of NfL for each MVPA (B) and CRF (D) quartile, respectively. CRF, cardiorespiratory fitness; MVPA, moderate‐to‐vigorous physical activity; NfL, neurofilament light chain.

Higher MVPA was significantly associated with lower concentrations of NfL (β = −0.107; 95% CI = −0.192, −0.023; *p* = 0.013; Figure 1A) and higher concentrations of p‐tau181 (β = 0.094; 95% CI = 0.010, 0.177; *p* = 0.028), adjusting for age, sex, waist circumference, *APOE* ε4, and study site (Table 2). When excluding one participant with 0 minutes of MVPA, associations with NfL persisted (β = −0.110; 95% CI = −0.195, −0.025; *p* = 0.011) while the association with p‐tau181 was trending but non‐significant (β = 0.083; 95% CI = −0.0031, 0.167; *p* = 0.051). Sex, *APOE* ε4, and Aβ positivity did not moderate observed associations.

**TABLE 2:** Associations between MVPA and cardiorespiratory fitness with brain and fluid measures of AD‐related pathology.

	Cardiorespiratory fitness			MVPA		
Outcome	β	95% CI	*p*	β	95% CI	*p*
NfL, pg/mL	−0.114	−0.215 to −0.013	0.027	−0.107	−0.192 to −0.023	0.013
GFAP, pg/mL	−0.063	−0.156 to 0.03	0.181	−0.037	−0.115 to 0.041	0.349
p‐tau181, pg/mL	0.071	−0.029 to 0.171	0.164	0.094	0.01 to 0.177	0.028
p‐tau217, pg/mL	0.063	−0.037 to 0.163	0.214	0.039	−0.044 to 0.121	0.361
Aβ1–42/1–40 ratio	−0.028	−0.133 to 0.077	0.604	0.007	−0.08 to 0.094	0.874
PET Centiloid	0.125	−0.01 to 0.259	0.07	−0.047	−0.156 to 0.063	0.402

*Notes*: Results of linear regression analyses adjusted for age, sex, waist circumference, *APOE* ε4, and study site. Reported as standardized β and 95% confidence intervals, in which both predictor and outcome variables were *z* scored. Skewed variables (MVPA, p‐tau217, p‐tau181, NfL, GFAP) were log‐transformed prior to *z* score standardization.

Abbreviations: Aβ, amyloid beta; AD, Alzheimer's disease; *APOE*, apolipoprotein E; CI, confidence interval; GFAP, glial fibrillary acidic protein; MVPA, moderate‐to‐vigorous physical activity; NfL, neurofilament light chain; PET, positron emission tomography; p‐tau, phosphorylated tau.

To further examine these relationships, post hoc analysis of covariance models tested MVPA quartiles and showed consistent associations with NfL (*F*[3,542] = 3.692; *p* = 0.012). Pairwise comparisons across quartiles showed that those in the lowest quartile of MVPA (reflecting < 12 minutes of daily MVPA) drove the association with higher NfL concentrations (Figure 1B).

MVPA was not significantly associated with Aβ PET Centiloid, GFAP, Aβ1–42/1–40 or p‐tau217 (Table 2). In logistic regression models, MVPA was not associated with Aβ positivity using either the p‐tau217 cut‐off (odds ratio [OR] = 1.07; 95% CI = 0.855, 0.134; *p* = 0.569) or PET Centiloid (OR = 0.861; 95% CI = 0.597, 1.250; *p* = 0.427). Results persisted in sensitivity analyses excluding participants with < 4 or > 10 valid days of accelerometer data (*N* = 30).

**FIGURE 2:**
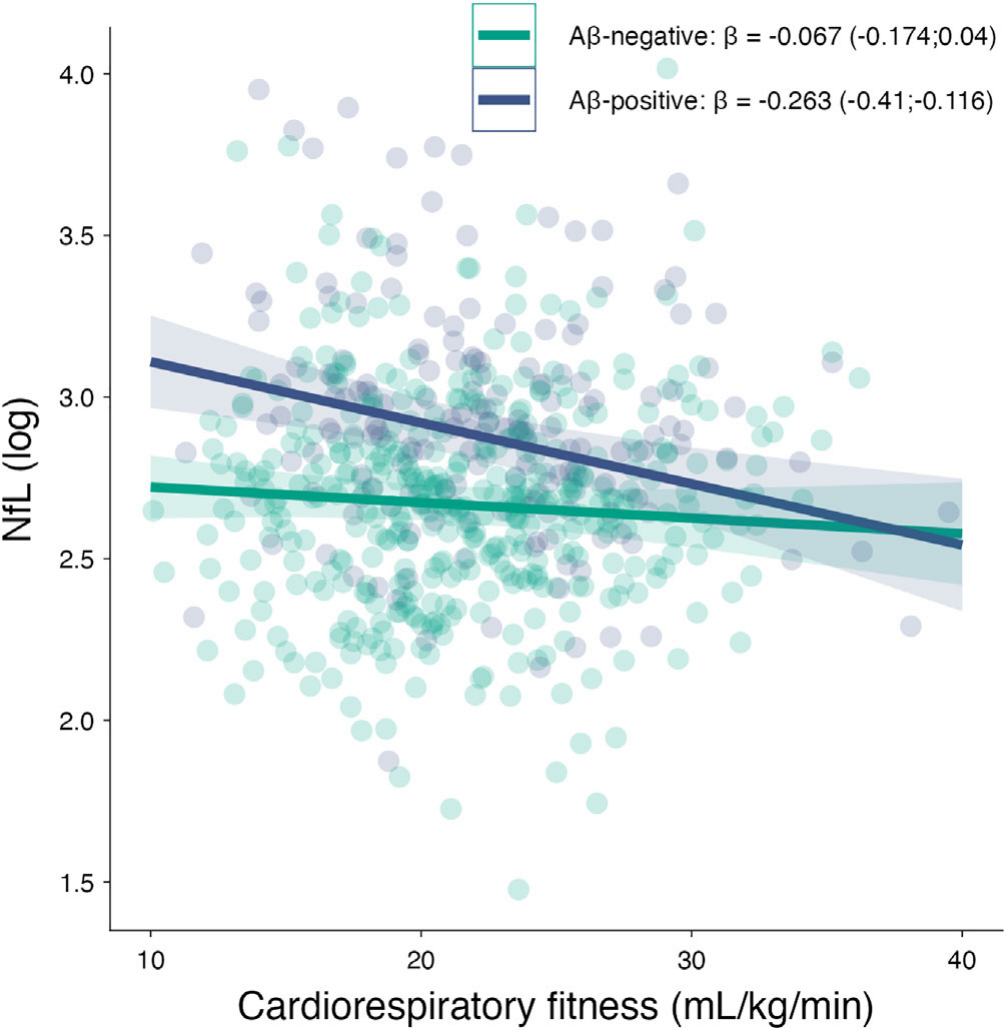
Moderating role of Aβ positivity in the relationship between CRF and NfL. Linear regression results demonstrating moderation of the relationship between CRF and NfL by Aβ positivity, defined using a clinical cutoff of p‐tau217 ≥ 0.46 pg/mL. Values (top right) represent standardized β values from simple slope analysis and shaded areas reflect 95% confidence intervals. Aβ, amyloid beta; CRF, cardiorespiratory fitness; NfL, neurofilament light chain; p‐tau, phosphorylated tau.

Similar to MVPA, higher CRF was significantly associated with lower concentrations of NfL (β = −0.114; 95% CI = −0.215, −0.013; *p* = 0.027; Figure 1C). Post hoc analysis of covariance models examining CRF quartiles showed consistent associations (*F*[3,595] = 3.223; *p* = 0.022), with those in the lowest CRF quartile demonstrating significantly higher NfL concentrations relative to those in the three upper quartiles (Figure 1D). This relationship was significantly moderated by Aβ positivity (Figure 2; *p* for int = 0.015), such that the association between higher CRF and lower NfL was specific to those classified as Aβ positive per the p‐tau217 cutoff (≥ 0.46 pg/ml; *N* = 181; β = −0.263; 95% CI = −0.410, −0.116; *p* < 0.001) compared to Aβ‐negative participants (*N* = 444; β = −0.067; 95% CI = −0.174, 0.040; *p* = 0.221). Sex and *APOE* ε4 did not moderate observed associations. In a post hoc exploratory analysis in the subsample with PET imaging, moderation by amyloid positivity defined as Centiloid ≥ 24 (Aβ positive *N* = 61) was non‐significant (*p* = 0.913).

CRF was not significantly associated with Aβ PET Centiloid or remaining blood biomarkers (Aβ1–42/1–40, p‐tau181, p‐tau217, GFAP). In logistic regression models, CRF was not associated with Aβ positivity using either the p‐tau217 cut‐off (OR = 1.02; 95% CI = 0.975, 1.07; *p* = 0.375) or PET Centiloid (OR = 1.08; 95% CI = 0.999, 1.170; *p* = 0.057).

### 3.3 Associations among MVPA, fitness, AD‐related biomarkers, and cognition

**FIGURE 3:**
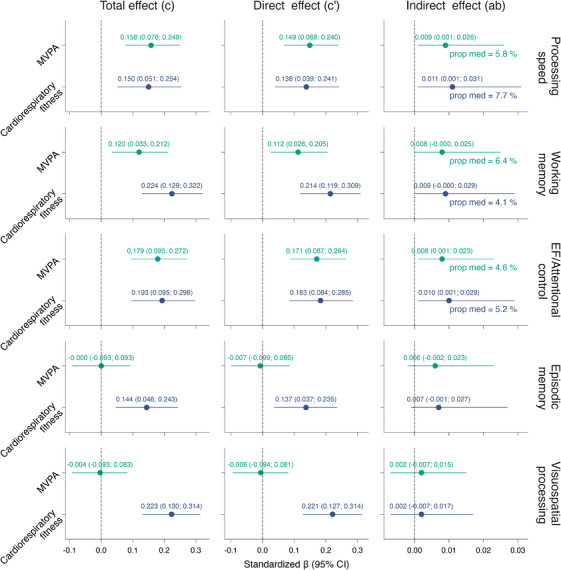
Mediation models examining indirect associations between MVPA, CRF, and cognition via NfL. Results of statistical mediation models testing whether NfL mediated associations between MVPA (green) or CRF (blue; predictors) and cognitive performance (outcome). Mediation models controlled for age, sex, years of education, waist circumference, *APOE* ε4, and study site. Dot and whisker plots represent standardized β values and 95% CIs, respectively. The proportion mediated was calculated as the ratio of the indirect and total effect and is shown only for cognitive domains demonstrating trending or significant indirect effects. *APOE*, apolipoprotein E; CI, confidence interval; CRF, cardiorespiratory fitness; EF, executive function; MVPA, moderate‐to‐vigorous physical activity; NfL, neurofilament light chain.

We further examined whether biomarkers associated with MVPA and CRF statistically mediated relationships with cognitive performance. Higher MVPA was significantly associated with better performance in the domains of EF/attentional control (β = 0.179, *p* < 0.001), working memory (β = 0.120, *p* = 0.011), and processing speed (β = 0.158, *p* < 0.001), adjusting for age, sex, years of education, waist circumference, *APOE* ε4 carriership, and study site. In statistical mediation models, NfL significantly mediated the association between MVPA and cognitive performance in the domains of processing speed (indirect effect β = 0.009; 95% CI = 0.001, 0.026, proportion mediated = 5.8%) and EF/attentional control (indirect effect β = 0.008; 95% CI = 0.001, 0.023, proportion mediated = 4.6%), with a trending indirect association observed for working memory (indirect effect β = 0.008; 95% CI = −0.001, 0.025, proportion mediated = 6.4%; Figure 3).

As described in our previous analysis of this sample, higher CRF was significantly associated with better performance across all five cognitive domains.^26^ NfL significantly mediated the association between CRF and processing speed (indirect effect β = 0.011; 95% CI = 0.001; 0.031, proportion mediated = 7.7%) and EF/attentional control (indirect effect β = 0.010; 95% CI = 0.001, 0.029, proportion mediated = 5.2%). A similar, though trending, indirect association was observed for working memory (indirect effect β = 0.009; 95% CI = −0.001, 0.029, proportion mediated = 4.1%; Figure 3). Thus, higher MVPA and CRF were associated with lower NfL which, in turn, related to better cognitive performance. NfL did not mediate associations with episodic memory or visuospatial processing. P‐tau181 did not statistically mediate associations between MVPA or CRF and cognitive performance in any domain.

## 4 DISCUSSION

In this study, higher MVPA and CRF were associated with lower concentrations of NfL, a general marker of neurodegeneration, but not measures of core AD pathology in a large cohort of cognitively unimpaired older adults. NfL was a significant statistical mediator of the relationships between MVPA, CRF, and performance in the EF/attentional control and processing speed domains, highlighting this as a potential pathway linking PA and fitness to preservation of cognitive function in older adulthood. In addition, the relationship between CRF and NfL was specific to Aβ‐positive individuals, indicating that maintaining higher fitness may help mitigate neurodegenerative changes in the setting of preclinical AD pathology. This study advances prior research in several ways. In addition to obtaining objective measures of both PA and aerobic capacity, we examined a panel of biomarkers reflecting multiple pathophysiological mechanisms of AD including those scarcely assessed in prior work (e.g., p‐tau217, GFAP), and evaluated associations with cognition across diverse cognitive domains.

NfL emerged as the primary biomarker associated with both MVPA and CRF. These findings suggest that the neuroprotective benefits of MVPA may be conferred through mechanisms influencing neurodegeneration, and expand on two recent studies in cognitively unimpaired older adults demonstrating inverse associations between self‐reported PA and plasma NfL concentrations.^15^, ^21^ Moreover, in clinical populations including autosomal dominant frontotemporal dementia and multiple sclerosis, greater self‐reported PA has been related to lower NfL levels, slower increases in NfL over time, and reduced concentrations of NfL after acute exercise.^18^, ^19^ Converging evidence from neuroimaging studies further supports these associations, as greater PA has been linked to less regional and total brain atrophy and preservation of white matter microstructure,^48^, ^49^, ^50^, ^51^ and exercise trials have found that aerobic training increases cortical volume and preserves hippocampal morphology.^52^, ^53^, ^54^ The mechanisms linking MVPA and fitness to neurodegeneration are likely multifaceted. In animal models, PA positively influences multiple pathways related to neurodegeneration including neuroinflammation, insulin signaling, cerebral perfusion, endothelial function, synaptogenesis, and neurogenesis,^30^, ^55^ though translational work in humans is needed to better characterize these pathways.

The relationships between greater amounts of MVPA and higher CRF to lower NfL were observed regardless of *APOE* ε4 status and sex. Importantly, females and *APOE* ε4 carriers are at elevated risk for AD, and our findings indicate that higher MVPA and fitness may benefit neurodegenerative pathways even among those at greater risk for decline.

PA and CRF showed no associations with brain Aβ, p‐tau217, Aβ1–42/1–40, or GFAP. These results indicate that MVPA and CRF, at least in the ranges captured in this study, are not cross‐sectionally related to variation in markers of AD‐specific pathology. While causal evidence is needed, it is possible that greater MVPA and higher fitness may support cognitive health by facilitating broader neuroprotective processes, including those influencing neurodegeneration, rather than through AD‐specific pathways. Consistent with this, most observational research has failed to find associations between PA and brain and fluid measures of amyloid pathology,^6^, ^24^ and several pilot intervention studies report no effects of 4 to 6 months of PA training on core AD biomarkers.^9^, ^10^, ^56^ However, discrepancies remain with several studies reporting inverse relationships between PA and AD pathology.^7^, ^8^, ^20^ Notably, one prior study examining PA associations with p‐tau217 and GFAP found that relationships with self‐reported PA were observed in those with cognitive impairment (mild cognitive impairment, AD) but not cognitively unimpaired older adults,^20^ suggesting that these associations might differ across the disease continuum. Here, we sought to examine associations with emerging pathology in those without clinical impairment, a point at which targeted interventions may be most efficacious. However, longitudinal studies encompassing individuals at various biological and clinical stages of AD will be vital for understanding the temporality of these relationships and how this may evolve across disease stages.

A major finding of this study was that Aβ status moderated the relationship between CRF and NfL, with higher CRF related to lower NfL specifically among Aβ‐positive individuals. These results suggest that maintaining higher fitness may confer resilience against neurodegenerative changes in the setting of preclinical AD pathology. Prior studies have found that higher fitness and PA mitigate the cognitive impact of AD pathology, attenuating associations between AD pathology and dementia risk and predicting slower rates of decline in dementia‐free older adults with high pathologic burden.^22^, ^57^ Our findings support the notion that fitness‐related variability in neurodegenerative processes may contribute to cognitive resilience in the presence of emerging AD pathology. A post hoc exploratory analysis using Centiloid‐defined Aβ status as a moderator was null, but restricting analyses to the PET subsample substantially reduced power to detect associations. Nevertheless, additional research is needed to confirm these findings. If replicated, future work should examine whether PA may serve as a stochastic factor—mitigating downstream neurodegenerative changes resulting from the AD cascade—or whether PA may modulate multiple pathways separate from the AD cascade to preserve brain health (e.g., by supporting synaptogenesis, vascular health, and enhancing the efficiency or flexibility of brain networks to compensate for AD‐related changes).

NfL statistically mediated associations between MVPA, CRF, and cognitive performance in the domains of EF/attentional control and processing speed, highlighting this as a potential pathway underlying the cognitive benefits of higher fitness and MVPA. A trending indirect association was also observed for working memory. Our findings complement and expand on a recent study showing that NfL significantly mediated the positive association between higher self‐reported PA and performance on a cognitive screening measure (Mini‐Mental State Examination).^20^ In the present study, cognitive performance was measured with more granularity and precision than prior work, using latent factors derived from > 15 cognitive measures to reflect multiple cognitive domains. Notably, the domain‐specificity observed here aligns with the cognitive processes showing the most consistent benefits with aerobic training in exercise intervention trials.^58^

A strength of this study is the use of two objective measures related to PA—MVPA measured via actigraph to classify PA behaviors and VO_2peak_ to capture CRF, a physiological marker of aerobic capacity. Notably, CRF is strongly linked to morbidity, mortality, and dementia risk in older adulthood,^30^ and can be modified by participation in regular exercise,^59^ though only one prior study has examined CRF associations with AD blood biomarkers.^9^ We observed correspondence in biomarker‐specific relationships between MVPA and CRF, indicating that both PA behavior and a modifiable physiological indicator of aerobic capacity were related to a blood biomarker of neurodegeneration that mediated associations with cognitive performance. Notably, CRF as a measure reflects several physiological pathways relevant to aerobic capacity (e.g., cardiovascular, pulmonary, metabolic), and the specific physiological processes that contribute to CRF and that may impact these biomarkers warrants further exploration.

There was an unexpected positive relationship between MVPA and p‐tau181. One prior study failed to find a cross‐sectional association between self‐reported MVPA and p‐tau181,^17^ while another reported a trending positive association with p‐tau181^22^ similar to the present findings. In this study, MVPA was unrelated to several biomarkers with greater accuracy for reflecting AD pathology than p‐tau181 (p‐tau217, PET brain Aβ), suggesting this association may be attributable to peripheral rather than central sources of p‐tau181. Moreover, the relationship between MVPA and p‐tau181 was trending but non‐significant after excluding one participant with zero daily MVPA, suggesting this individual may have driven the observed association and that caution is needed in interpreting this result.

The present study has several limitations. The analyses were cross‐sectional and causal inferences are therefore limited. While higher MVPA and fitness may influence NfL, it is also possible that those with less neurodegeneration are able to maintain higher fitness levels and MVPA engagement. Additional insight will be gained from prospective studies examining trajectories of change and intervention trials manipulating PA levels to examine effects on blood biomarkers. Notably, this study focused on AD markers of amyloidosis, though tau pathology is more closely linked to neurodegeneration.^60^ Future studies capturing and isolating tauopathy will be important for determining whether tau status may also moderate fitness relationships to neurodegenerative changes. The sample was highly educated, with a higher proportion of females and composed of adults aged 65 to 80 years. Age is a robust predictor of disease pathogenesis, and our results should be interpreted within the age range examined here. Other studies are needed to replicate our findings across varied populations, including a greater proportion of males, individuals with cognitive impairment, various levels of education, and a broader age range to capture those in the ninth and tenth decades of life. Additionally, self‐selection into a clinical trial such as IGNITE may reflect individual factors that could limit generalizability to the broader population. Notably, however, the frequency of amyloid abnormality and distribution of blood biomarkers align with studies in other similarly aged cognitively unimpaired cohorts,^11^, ^61^, ^62^ suggesting our sample was not biased with regard to pathologic burden.

While the sample demographics matched the population demographics of the surrounding community for each site, including 24% of individuals from racial and ethnic minority groups, meeting US Census–based recruitment targets may be insufficient to infer generalizability across diverse populations. While study participants demonstrated a range of PA and fitness levels, participants were considered relatively inactive at the time of enrollment. While this is broadly representative of the general aging population, with two thirds of US older adults not meeting national PA recommendations,^63^ the lack of highly active individuals may have influenced observed associations. However, these findings suggest that even small differences in MVPA and fitness may have important relationships with neurodegenerative pathways. In addition, if replicated, even small effects can have large public health implications at the population level.

In conclusion, higher MVPA and fitness may confer cognitive benefits through mechanisms influencing neurodegeneration rather than AD‐specific pathways in cognitively unimpaired older adults, though confirmation from longitudinal studies examining the trajectory of PA associations with AD‐related pathology is needed. In addition, maintaining higher fitness may mitigate neurodegenerative changes in individuals with emerging AD pathology, highlighting this as a promising modifiable target for early intervention.

## CONSENT STATEMENT

The study was approved by the institutional review board at each site with the following protocol numbers: Pittsburgh (STUDY19110244), Kansas (STUDY00140896), and Northeastern (17‐05‐02). All participants provided written informed consent before data collection.

## CONFLICT OF INTEREST STATEMENT

The authors declare no conflicts of interest relevant to this manuscript. Kirk I. Erickson consults for MedRhythms, Inc., and Neo Auvra, Inc. John M. Jakicic is on the scientific advisory board for Wondr Health, Inc. Renee J. Rogers is a consultant for Seca and Wondr Health, Inc., and is on the advisory board for AstraZeneca and Neurocrine Biosciences. Thomas K. Karikari is an inventor on a University of Pittsburgh patent regarding the IPMS assay for Aβ peptides. Thomas K. Karikari serves a consultant for Quanterix, outside the submitted work. Lauren E. Oberlin, Patricio Solis‐Urra, Kelsey R. Sewell, Audrey M. Collins, Chaeryon Kang, Haiqing Huang, George Grove, Lu Wan, Arthur F. Kramer, Edward McAuley, Jeffrey M. Burns, Charles H. Hillman, Eric D. Vidoni, Anna L. Marsland, M. Ilyas Kamboh, Amanda Szabo‐Reed, and Jill Morris have no disclosures. Author disclosures are available in the supporting information.

## Supporting information



Supporting Information: alz71484‐sup‐0001‐TableS1.docx

Supporting Information: alz71484‐sup‐0002‐Disclosureforms.pdf
