# Supplementary material for: Physical activity, aerobic fitness, and AD blood biomarkers: The IGNITE study
Source: Alzheimers Dement. 2026 May 18;22(5):e71484. doi: 10.1002/alz.71484 (PMC13183585; doi:10.1002/alz.71484)
Supplement: Supplementary file 1 — Supporting Information: alz71484‐sup‐0001‐TableS1.docx [file ALZ-22-e71484-s002.docx]

Supplementary Table 1. Descriptive Characteristics of Full IGNITE Cohort and Stratified by Amyloid Status.

|  | | | | |
| --- | --- | --- | --- | --- |
|  | **Overall** | **Amyloid Status**^2^ | | |
| **Variable** | **N = 648**^1^ | **Aβ-negative**  N = 444 | **Aβ-positive**  N = 181 | **p-value**^3^ |
| Age, yr | 69.9 ± 3.7 | 69.4 ± 3.6 | 70.9 ± 3.9 | <0.001 |
| Education, yr | 16.32 ± 2.21 | 16.24 ± 2.24 | 16.47 ± 2.13 | 0.2 |
| Waist circumference, cm | 98 ± 14 | 99 ± 14 | 98 ± 15 | 0.5 |
| Cardiorespiratory fitness (m​L/kg/min) | 21.7 ± 5.1 | 21.6 ± 5.0 | 22.3 ± 5.2 | 0.11 |
| MVPA (min/day) | 31 ± 26 | 32 ± 26 | 31 ± 27 | >0.9 |
| Race, white *N* (%) | 491 (76%) | 328 (74%) | 152 (84%) | 0.007 |
| *APOE4*-carriage |  |  |  | <0.001 |
| APOE4 non-carrier, *N* (%) | 466 (73%) | 349 (79%) | 108 (60%) |  |
| APOE4 carrier, *N* (%) | 174 (27%) | 95 (21%) | 73 (40%) |  |
| Sex, *N* % |  |  |  | 0.006 |
| Female | 461 (71%) | 329 (74%) | 114 (63%) |  |
| Male | 187 (29%) | 115 (26%) | 67 (37%) |  |
| Site, *N* % |  |  |  | 0.4 |
| Kansas | 214 (33%) | 149 (34%) | 63 (35%) |  |
| Northeastern | 215 (33%) | 142 (32%) | 65 (36%) |  |
| Pitt | 219 (34%) | 153 (34%) | 53 (29%) |  |
| Episodic memory | 0.00 ± 0.61 | 0.04 ± 0.60 | -0.07 ± 0.64 | 0.045 |
| Processing speed | 0.00 ± 0.62 | 0.04 ± 0.62 | -0.09 ± 0.63 | 0.020 |
| Working memory | 0.00 ± 0.54 | 0.03 ± 0.54 | -0.04 ± 0.54 | 0.2 |
| EF/attentional control | 0.00 ± 0.59 | 0.02 ± 0.60 | -0.05 ± 0.58 | 0.2 |
| Visuospatial processing | 0.00 ± 0.56 | 0.00 ± 0.57 | 0.03 ± 0.56 | 0.6 |
| NfL, pg/mL | 17 ± 7 | 15 ± 6 | 20 ± 8 | <0.001 |
| GFAP, pg/mL | 179 ± 97 | 163 ± 83 | 216 ± 116 | <0.001 |
| pTau181, pg/mL | 2.83 ± 1.55 | 2.39 ± 1.15 | 3.87 ± 1.86 | <0.001 |
| pTau217, pg/mL | 0.43 ± 0.28 | 0.30 ± 0.08 | 0.77 ± 0.31 | <0.001 |
| Aβ1-42/1-40 ratio | 0.099 ± 0.013 | 0.101 ± 0.012 | 0.096 ± 0.014 | <0.001 |
| Centiloid value | 8 ± 30 | -2 ± 14 | 32 ± 40 | <0.001 |
| Note: Unless otherwise specified, data are presented as mean ± standard deviation. Abbreviations: NfL, neurofilament light chain; GFAP, glial fibrillary acidic protein; p-tau, phosphorylated tau; Aβ, amyloid beta; MVPA, moderate-to-vigorous physical activity; APOE, apolipoprotein E. | | | | |
| ^1^As previously reported, the biomarker sample sizes varied slightly due to different amounts of missing data and outliers for each biomarker. Eight subjects did not have APOE genotype data and 59 subjects did not have accelerometry data (MVPA).  ^2^A pTau217 threshold of 0.46 pg/mL was used to classify Aβ-positivity. This cut-point was established in this sample using Area Under the Curve analysis and the Youden index, as previously described | | | | |
| ^3^Welch Two Sample t-test; Pearson's Chi-squared test. Significant values (*p* < 0.05) reflect variables that differ between the Aβ-positive and Aβ-negative groups. | | | | |
